# Study on Residual Stress in Disc-Milling Grooving of Blisks

**DOI:** 10.3390/ma15207261

**Published:** 2022-10-18

**Authors:** Hongmin Xin, Taotao Xing, Hui Dai, Jian Zhang, Changfeng Yao, Minchao Cui, Qinggui Zhang

**Affiliations:** 1 Hubei Key Laboratory of Power System Design and Test for Electrical Vehicle, Hubei University of Arits and Science, Xiangyang 441053, China; 2 Key Laboratory of Aero-Engine High Performance Manufacturing, Ministry of Industry and Information Technology, Northwestern Polytechnical University, Xi’an 710072, China; 3 Hubei Chaozhuo Aviation Technology Co., Ltd., Xiangyang 441100, China

**Keywords:** blisk, disc-milling, grooving, residual stress

## Abstract

The disc-milling method is expected to increase the grooving efficiency of blisks. However, there are few studies about the residual stress on a blisk during disc-milling grooving. In this study, a single-factor experiment and an orthogonal experiment of blisk disc-milling and grooving were designed to obtain the residual stress. Surface subsurface residual stress were also studied. The results showed that the surface of the milling groove bore compressive stress. Residual stress decreased with increasing spindle speed and increased with increasing feed speed and spindle rotation angle. Moreover, residual stress was most sensitive to spindle rotation angle and least sensitive to feed speed. A higher residual stress produced on the machined surface led to a deeper layer of residual stress.

## 1. Introduction

With the rapid development of China’s aviation industry, the overall performance requirements of aircraft engines have also increased. As a key component of aircraft engines, integral blisks can effectively reduce their overall weight and improve aerodynamic performance. However, due to the complex structure, narrow channel, and poor openness of the overall blisk, it is difficult to manufacture and process it, which has become a major technical problem in the aviation field. In recent years, a series of manufacturing techniques have been explored, such as milling [1], electrical discharge machining [2], electrolytic machining [3], laser cladding [4], and other techniques to manufacture blisks. Among them, milling has the advantages of having high reliability and a small production cycle and has been widely used in the field of blisk manufacturing [5]. Disc-milling is the first process of grooving the whole blisk. The blank material of the blisk is cut with a large margin, and the whole blisk is processed in cooperation with other technologies.

Titanium alloys have been widely used in the aviation field due to their high specific strength, low thermal conductivity, and good high-temperature performance, such as aircraft engine integral blisks. At present, scholars at home and abroad have carried out active research on residual stress in titanium alloy milling and proposed many theories and methods. Residual stress can affect the surface integrity of the workpiece. Zhang et al. [6] proposed an ultrasonic longitudinal–torsional composite milling method to obtain large residual compressive stress on the surface of the workpiece and improve the surface integrity and fatigue life of the workpiece. The influence of different process parameters on the surface residual stress of titanium alloy thin-walled parts was studied. Finally, the experimental results show that the method is effective. In order to study the effect of residual stress on the hardness value of weld metal, Terentyev et al. [7] studied and analyzed titanium alloy welded joints obtained by electron beam welding and argon arc welding. The results showed that the nature of residual stress distribution depends on welding parameters and can affect the hardness value of titanium alloys in welds. When machining titanium alloy blades, abrasive belt grinding can prolong the fatigue life of the blade, but it will also affect its performance. Xiao et al. [8] used ABAQUS to establish a simulation model of titanium alloy grinding and analyzed the effect of grinding process parameters on the action law of surface residual stress; the final simulation results are consistent with the actual experiment results. Kaifa et al. [9] established 3D finite element models associated with two original surface roughnesses as well as without original surface roughness based on Gaussian distribution combined with exponential autocorrelation function and performed integrated coupled DEM–FEM simulations of the shot peening process for these three target models to study shot peening residual stresses. The results showed that the shot peening surface residual stresses with the presence of original surface roughness were more uniformly distributed. In titanium alloy additive manufacturing, high residual stresses can exist in the sample due to layer-by-layer fabrication, which can affect the fatigue strength of the product. Karpenko et al. [10] proposed a numerical method to predict the effect of residual stresses on the fatigue crack expansion rate (FCGR) and verified the validity of the method experimentally. Kyaramyan et al. [11] proposed several parameters for the combined surface heat treatment and air shot peening machining process of VT41 titanium alloy compressor impeller blades for advanced engines and analyzed the residual stresses in the initial state of the blades and after finishing treatment to derive the effects of each parameter on the microstructure and life of the blades. During titanium milling, the effect of thermal–mechanical coupling on the surface integrity of titanium alloy was studied [12].

During the machining process of blisk disc-milling grooving, the milling force is large, and the milling temperature is high. Due to the thermal–mechanical coupling effect, the machined surface and the subsurface will inevitably undergo mechanical, physical, and chemical changes; thus, a deep plastic deformation surface layer is formed on the machined surface. The working environment of the blisk is extremely harsh, and its surface quality requirements are very high. The size and direction of the surface residual stress and the depth of the residual stress layer affect the fatigue life of the blisk and, thus, affect the safety performance of the engine [13,14]. At present, scholars at home and abroad have carried out relevant research on the residual stress of the blisk. In order to study the influence of alumina ACW (abrasive cloth wheel) polishing parameters on the surface residual stress of a GH4169 superalloy blisk blade, Lin [15] obtained the residual stress prediction model of the polishing surface based on Minitab software and obtained the optimal range of process parameters, which can be used to obtain large and stable surface residual compressive stress. Alcaraz et al. [16] used a new horizontal vibration shot peening method to understand the combined shot peening and polishing mechanism during the vibration shot peening a process of the three-stage blisk of a gas turbine engine. They achieved a simultaneous reduction in overall production cost and time of vibratory peening for all three stages of blisks. In order to better study the residual stress of aircraft engine blades, Xian Chao et al. [17] established a simple mathematical model of residual stress based on the force effect, thermal effect, and the coupled effect of force and heat and verified the accuracy of the model by experiments. Wu et al. [18] established an empirical model of the surface residual stress based on the polishing process parameters and quantitatively analyzed the effect of the polishing process on deformation through finite element analysis and experimental testing. The results showed that this surface polishing process can effectively improve surface integrity.

It can be seen from the above that, although there have been some achievements in the related experimental research on the residual stress of titanium alloy materials and the residual stress of the overall blisk machining process at home and abroad, the above research concerns the residual stress under the processing technology of shot peening, polishing, and grinding; however, none of the studies involved the analysis of residual stress in the milling and grooving of the blisk. Therefore, the research on the residual stress of the high-efficiency grooving machining of the blisk disc-milling is carried out here, and the theoretical and practical basis for the elimination of the residual stress in the grooving machining of the disc-milling is provided, and it is of great significance to finally make the disc-milling and grooving of the blisk meet the technological requirements. In this paper, the titanium alloy TC17 blisk blank is used as the processing object, a single-factor experiment and an orthogonal experiment of blisk disc-milling and grooving are designed, the residual stress prediction model is established, and the residual stress on the surface of blisk disc-milling and grooving is carried out, analyzing the influence of process parameters on residual stress and providing theoretical support for improving the fatigue life of blisk parts.

## 2. Materials and Methods

### 2.1. Materials

TC17 was chosen as the experimental material in this study. It has many advanced properties such as good comprehensive mechanics performance, great specific strength, and low thermal conductivity, which extend its applications in aviation. The main chemical composition and mechanical properties are shown in Table 1 and Table 2, respectively.

### 2.2. Sample Preparation

**Figure 1:**
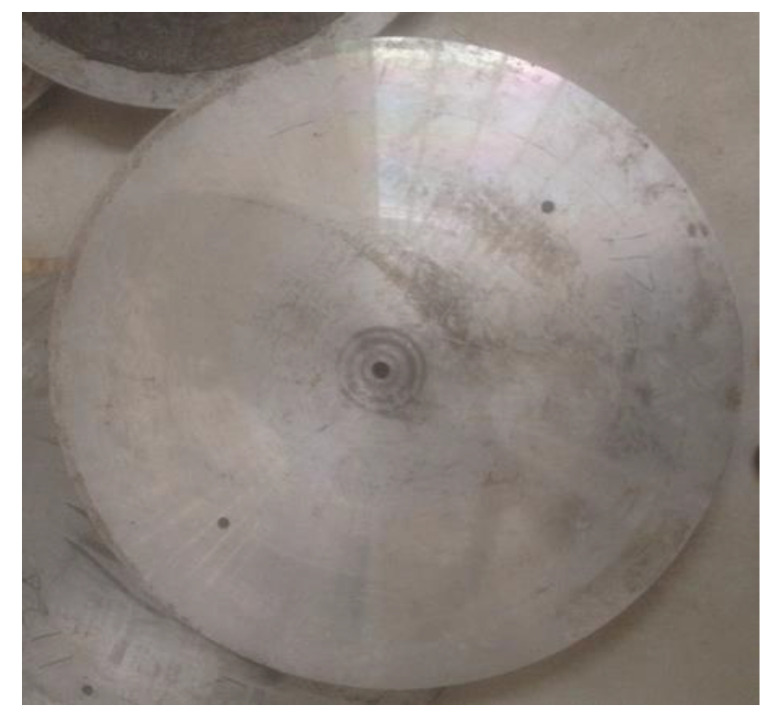
Integral blade plate blanks.

In order to show the real residual stress of the disc-milling process, one aircraft engine secondary compressor disc blank was prepared. Its forged part, with a size of Ø640 × 35 mm, is shown in Figure 1.

### 2.3. Disc-Milling Cutter

**Figure 2:**
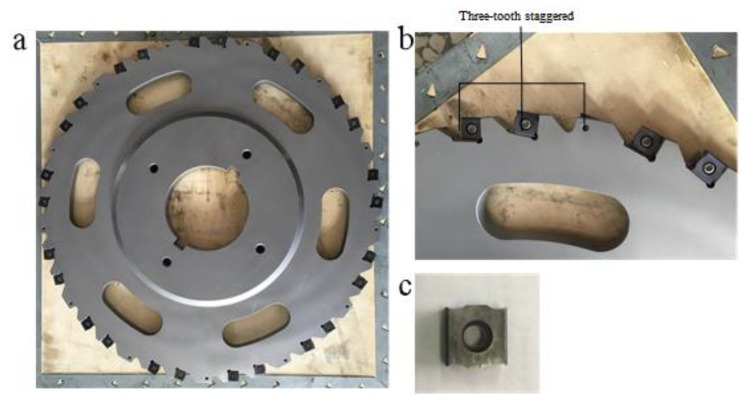
Disc-milling cutter disc, blade, and installation method. (**a**) Disc-milling cutter disc; (**b**) Blade installation method; (**c**) Blade.

In this experiment, a staggered-toothed disc cutter with three cutting edges, made by Zhuzhou Diamond Cutting Tool Co. Ltd. (Zhuzhou, China), was applied, as shown in Figure 2. The cutter was specially designed and manufactured on the basis of fully considering the structure of the blisk composite milling machine and the characteristics of the blisk channel. The tool was covered by an ultra-fine TiAlN nano-coating, which can not only effectively reduce the friction force of blade but also increase the toughness and hardness of the blade surface. The key parameters of the disc-milling cutter are shown in Table 3.

### 2.4. Residual Stress Test Method

**Figure 3:**
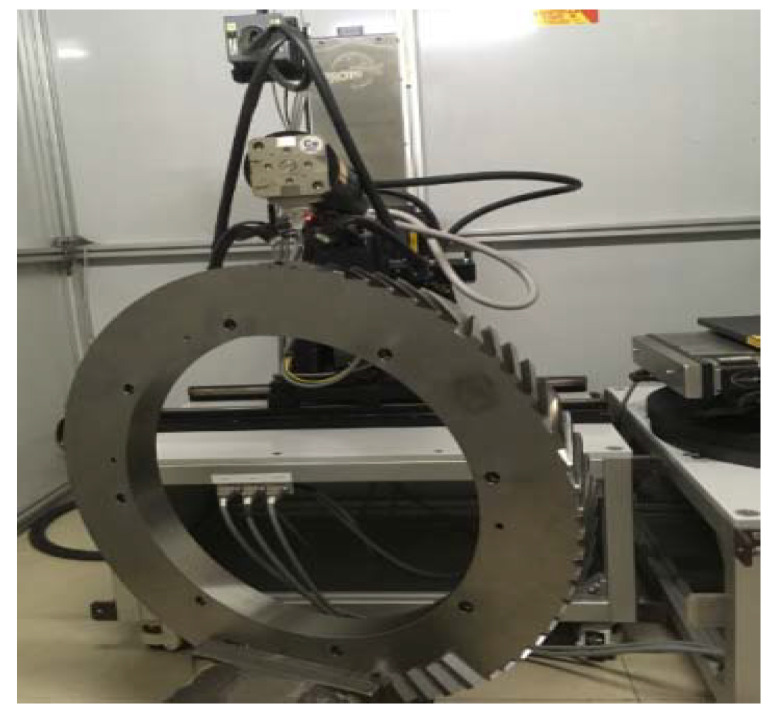
The scene for the residual stress of blisk test.

Residual stress was measured by using an XStress 3000 (PROTO Manufacturing, Oldcastle, ON, Canada) with CuKα radiation using the ψ-tilt X-ray method. Three equidistant points on the machined surface along the feed direction were examined. The mean values were calculated as the experimental value, which is denoted as σ. Figure 3 shows the scene for testing the residual stress of the blisk.

According to a previous study [12], the residual stress of a groove in a rectangular sample relates with spindle speed *n* (r/min), feed rate *v_f_* (mm/min), and discmilling head rotation angle *θ* (°). To investigate the influence of the above machining variables in disc-milling grooving on the surface residual stress of a blisk, an orthogonal experiment was designed. The selection of process parameters was determined according to the debugging experience and the performance parameters of the machine tool. The spindle speed was chosen as 30, 40, 50, and 60 (r/min). The feed rate was set to 15, 25, 35, and 45 (mm/min). The disc-milling head rotation angle was set to 25°, 35°, 45°, and 55°. The machining parameter configuration is shown in Table 4. Water and emulsified oil were, respectively, chosen as coolants to study the impact of the coolant on residual stress.

**Figure 4:**
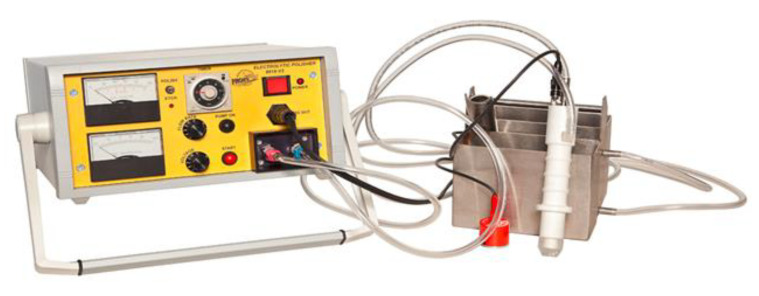
Electrolytic polishing machine.

To further find out the residual stress distribution of the subsurface, a single-factor experiment was carried out. The machining parameter configuration is shown in Table 5. An electrochemical method was used. The depth interval was chosen to be 10–20 µm. The residual stress was tested until close to zero. Figure 4 shows the electrolytic polishing machine used in the test.

## 3. Results and Discussion

### 3.1. Effect of Milling Parameters on Surface Residual Stress

**Figure 5:**
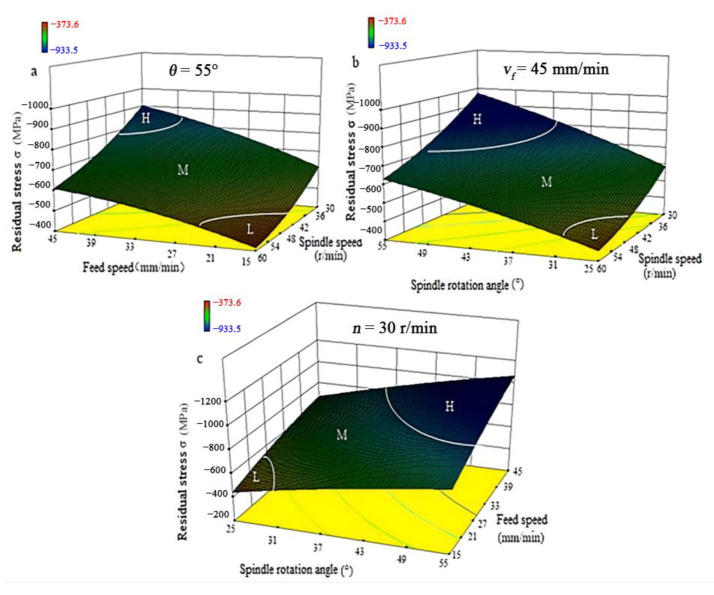
Surface residual stress using water as the coolant. (**a**) Surface residual stress versus feed speed and spindle; (**b**) Surface residual stress versus spindle rotate angle and spindle; (**c**) Surface residual stress versus spindle rotate angle and feed.

The residual stress is hard to plot in a three-dimension graph in that residual stress relates to three factors. To illustrate test results easily, two factors were used as the X and Y coordinates and residual stress was the Z coordinate, as shown in Figure 5. Since the residual stress test results follow the similar trend for different values of one factor, only results of *θ* = 55, *v_f_* = 45, and *n* = 30 are shown in Figure 5. In Figure 5, H represents residual stress below −800 MPa, M represents residual stress between −600 and −800 MPa, and L represents residual stress above −450 MPa. A minus sign ‘−’ represents compressive stress.

Figure 5 shows the residual stress while using water as the coolant. Figure 5 illustrates that the machined surface bears compressive stress. It can be seen from Figure 5a that the high-value region (H region) of residual stress *σ_w_* appears in the intersection region of high feed speed and low spindle speed, while the low-value region (L region) appears in the region where the low feed rate intersects with the high spindle speed. From Figure 5b, it can be seen that the H region appears in the intersection region of high spindle rotation angle and low spindle speed, while the L region appears in the region where the low spindle rotation angle intersects with the high spindle speed. Figure 5c shows that the H region appears in the intersection region of low spindle rotation angle and high feed speed, while the L region appears in the region where the high spindle rotation angle intersects with the low feed speed. Surface residual stress obeys the same trend as that for water when the coolant is emulsified oil. There are no detailed discussions about it.

**Figure 6:**
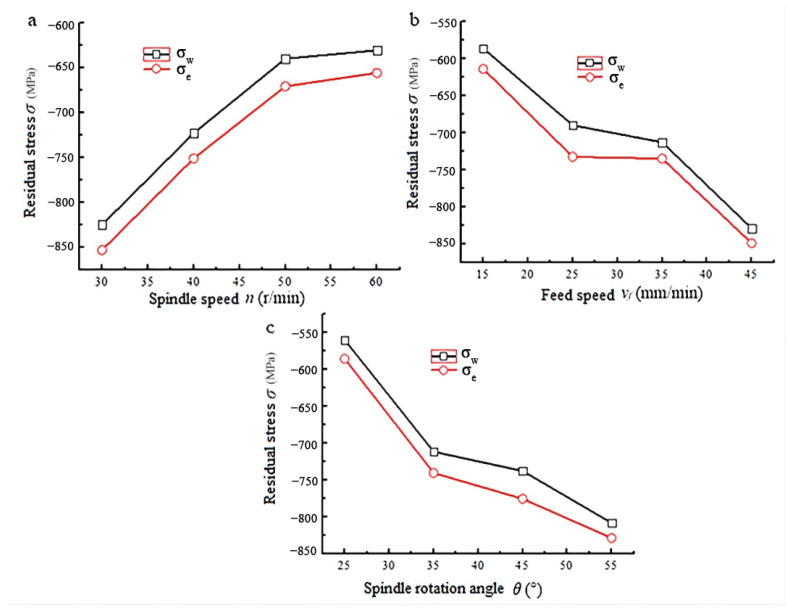
Influence of machining parameters on the surface residual stress processed. (**a**) Surface residual stress versus spindle; (**b**) Surface residual stress versus feed; (**c**) Surface residual stress versus spindle rotate.

To study the impact of the machining parameters on the surface residual stress further, range analysis was applied to process test data. In the range analysis method, a test value under one parameter equals the average value of test results under all other parameters. Figure 6 shows the relationship between machining parameters and surface residual stress processed. In Figure 6, it can be seen that surface residual stress when emulsion cooling (*σ_e_*) was used as coolant was larger than that when water (*σ_w_*) was used. It means that emulsion cooling is more suitable for the blisk disc-milling groove processing than water cooling, in that the residual compressive stress prolongs the fatigue life of parts. Moreover, the surface residual stresses *σ_w_* and *σ_e_* vary with machining parameters, following the same trend. The residual compressive stress decreases as spindle speed increases and increases with increasing feed speed and spindle rotation angle.

To investigate the sensitivity of surface residual stress to each machining parameter, multiple linear regression was used to describe the relationship between the original residual stress and the milling parameters, as shown in Equation (1). The ‘F’ method was applied to verify the validity of Equation (1). The results shown in Table 6 suggest that the ‘F’ value of *σ_w_* and *σ_e_* are all bigger than F_0.01_ (3, 12) and F_0.05_ (3, 12), which means the prediction effect is satisfactory. Equation (1) indicates that surface residual stresses *σ_w_* and *σ_e_* are most sensitive to spindle rotation angle and spindle speed secondary and least sensitive to feed speed.

(1) $$\left\{\begin{array}{c} \sigma_{w}=-10^{2.4569}n^{-0.3861}v_{f}{}^{0.2529}\theta^{0.4102}\\ \sigma_{e}=-10^{2.4198}n^{-0.3749}v_{f}{}^{0.2455}\theta^{0.3946} \end{array}\right.$$

### 3.2. Residual Stress Distribution of the Subsurface

**Figure 7:**
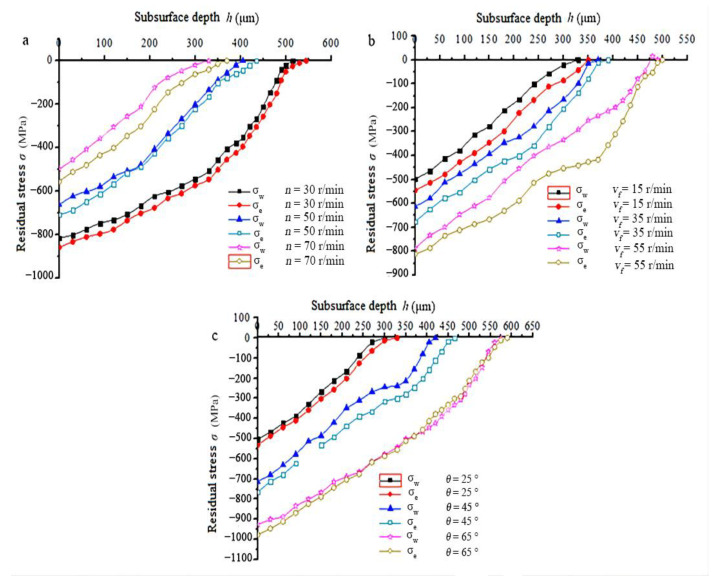
Residual stress of the subsurface. (**a**) Residual stress versus spindle; (**b**) Residual stress versus feed; (**c**) Residual stress versus spindle rotate.

The residual stress of each depth below the surface is plotted in Figure 7. It can be found that residual stress decreases as depth increases. In addition, depth of the residual stress layer, defined as the minimum depth of zone where residual stress is zero, when water was used was less than that when emulsified oil was used.

In Figure 7a, the feed speed and spindle rotation angle are constant, 35 mm/min and 45°, respectively. It can be found that the depth of the residual stress layer varies from 515 μm to 330 μm when the spindle speed changes from 30 r/min to 70 r/min for water cooling. It is indicated that depth of the residual stress layer increases as the spindle speed decreases. In Figure 7b, the spindle speed and spindle rotation angle are constant, 50 r/min and 45°, respectively. It can be found that the depth of the residual stress layer varies from 330 μm to 490 μm when the feed speed changes from 15 mm/min to 55 mm/min for water cooling. It is indicated that the depth of residual stress layer increases as the feed speed increases. In Figure 7c, the spindle speed and feed speed are constant, 50 r/min and 35 mm/min, respectively. It can be found that the depth of the residual stress layer varies from 300 μm to 575 μm when the spindle rotation angle changes from 25° to 65° for water cooling. It is indicated that the depth of the residual stress layer increases as the spindle rotation angle increases. The depth of the residual stress layer of emulsified oil cooling follows the same trend as that of water cooling.

**Figure 8:**
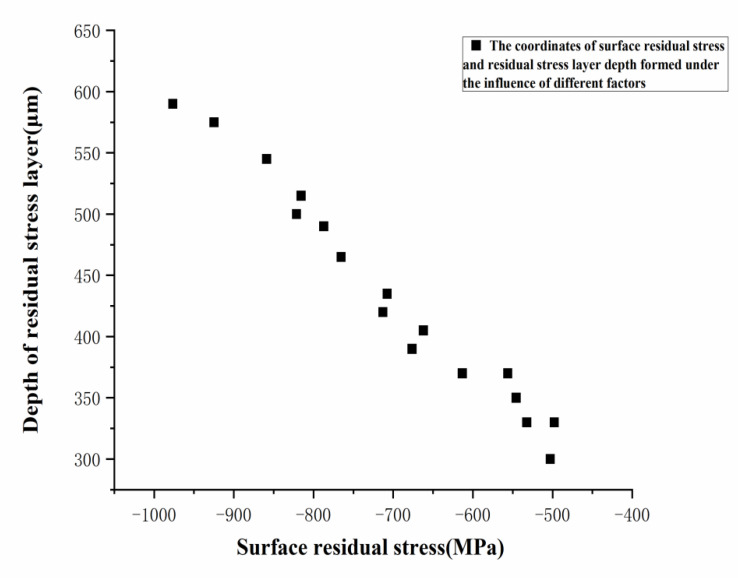
Depth of the residual stress layer versus surface residual stress.

A plot of the data of the depth of the residual stress layer and the surface residual stress in Figure 7 to the same figure, without consideration of the milling parameters, can be seen in Figure 8. It is indicated that a higher residual stress produced on the machined surface would lead to a deeper layer of residual stress.

The formation of the residual stress layer under the surface can be explained by the following factors. The large disc-milling force can induce severe plastic deformation between the flank of the cutting tools and the workpiece upon the skin layer due to significant friction and squeezing. Since the specific volume of the skin-layer metal increases and the volume expands under the processing condition, while the inner-layer metal prevents this behavior of the skin-layer metal, the residual compressive stress can thus be generated on the skin layer in the deformation zone [19]. High temperature in milling process is another important factor for compressive stress generation. Since TC17 has low heat conductivity and good heat plasticity, the milling heat is difficult to release when the contact length between the chip and rake face is short. The produced heat expands the volume of material around the skin layer, which also contributes to the generation of residual compressive stress [19]. Therefore, the distribution of residual stress is determined by the combined effect of the milling force and temperature. In fact, the residual compressive stress is profitable to improve the life of workpiece because fatigue cracking can be postponed or even prevented due to the existence of residual compressive stress [20,21].

## 4. Discussion

Since there are few reports at home and abroad on the residual stress of high-efficiency grooving machining of blisk disc-milling, the residual stress experiment designed for aero-engine blisk blanks in this paper can effectively make up for the gap in scientific research and guide the follow-up of blisk disc-milling. However, the disadvantage is that the single-factor experiments, orthogonal experiments, and linear regression techniques in the study are all based on previous research experience and achievements, and no new experimental methods and technologies have been explored, and there is no study focusing on the disc-milling and grooving of the blisk. Further in-depth research on machining residual stress failed to obtain the optimal method for solving residual stress and determining the optimal parameters for disc-milling. In future work, special experimental research should be done to address the shortcomings of the research, and based on the results obtained in this paper, the research on the processing optimization of blisk parts should be expanded, in order to improve the overall blisk fatigue life and various performances.

## 5. Conclusions

(1) The machined surface of the blisk disc-milling groove is under compressive stress, and the formation of residual stress is dominated by the milling force.
(2) The residual stress is most sensitive to the change of the rotation angle of the spindle, the influence of the spindle speed on the residual stress is second, and the effect of the feed speed on the residual stress is not obvious.
(3) The residual stress decreases with increasing spindle speed and increases with increasing feed speed and spindle rotation angle.
(4) The residual stress under emulsion oil cooling is greater than that of water cooling. A higher residual stress produced on the machined surface would lead to a deeper layer of residual stress.

## Figures and Tables

**Table 1:** Chemical composition of TC17 titanium alloy (mass fraction, %).

Elements	Al	Zr	Sn	Mo	Cr	Fe	C	N	H	O
Wt (%)	4.5~5.5	1.5~2.5	1.5~2.5	3.5~4.5	3.5~4.5	≤0.25	≤0.05	≤0.05	≤0.012	0.08~0.13

**Table 2:** Mechanical properties of TC17 titanium alloy (room temperature).

σ_b_ (Mpa)	σ_r_0.2 (Mpa)	δ (%)	ψ (%)
≥1180	≥1110	≥10	≥17.5

**Table 3:** Parameters of disc cutter.

Diameter (mm)	Tooth Number	Tool Thickness (mm)	Body Material	Blade Material	Rake Angle	Relief Angle	Tool Nose Radius (mm)	Inclination Angle
420	39	15	3Cr2MnNIMo	YBG212	20°	0°	0.8	0°

**Table 4:** Machining parameter configuration of orthogonal experiment.

No.	*n*(r/min)	*v_f_* (mm/min)	*θ* (°)
1	30	15	25
2	30	25	35
3	30	35	45
4	30	45	55
5	40	15	55
6	40	25	45
7	40	35	35
8	40	45	25
9	50	15	25
10	50	25	55
11	50	35	45
12	50	45	35
13	60	15	55
14	60	25	25
15	60	35	35
16	60	45	45

**Table 5:** Machining parameter configuration of single-factor experiment.

No.	*n* (r/min)	*v_f_* (mm/min)	*θ* (°)
1	30	35	45
2	50	35	45
3	70	35	45
4	50	15	45
5	50	35	45
6	50	55	45
7	50	35	25
8	50	35	45
9	50	35	65

**Table 6:** Test of significance of the residual stress model.

Parameter	Freedom	Quadratic Sum SS	Mean Square Sum MS	F Statistic	F_0.01_ (3, 12)	F_0.05_ (3, 12)
*σ_w_*	3	0.111343	0.037114	34.29	5.953	3.49
	12	0.012985	0.001082	/	/	/
	15	0.124328	/	/	/	/
*σ_e_*	3	0.104195	0.034732	55.86	5.953	3.49
	12	0.00746	0.000622	/	/	/
	15	0.111655	/	/	/	/
